# Use of biliopancreatic duct imaging system in a 13-month-old girl to enable endoscopic removal of a foreign body in the appendix

**DOI:** 10.1055/a-2308-2975

**Published:** 2024-05-17

**Authors:** Lujing Tang, Hong Zhao, Kerong Peng, Jingan Lou

**Affiliations:** 1 605254 Gastroenterology, Zhejiang University School of Medicine Children’s Hospital, Hangzhou, China; 2 National Clinical Research Center for Child Health, Hangzhou, China

A 13-month-old girl was admitted because of the observation over a month previously of a screw-like foreign body in the right lower quadrant. The patient was asymptomatic; physical examination showed normal findings. After admission, an X-ray showed a screw in the right lower quadrant ( Fig. 1 ). Our initial attempts to find the object by colonoscopy failed. Subsequently, a foreign body in the appendix was suspected, and ultrasound examination confirmed that this was indeed the case ( Fig. 2 ). After communication with the parents, they were first referred to endoscopic treatment for the child.

**Fig. 1:**
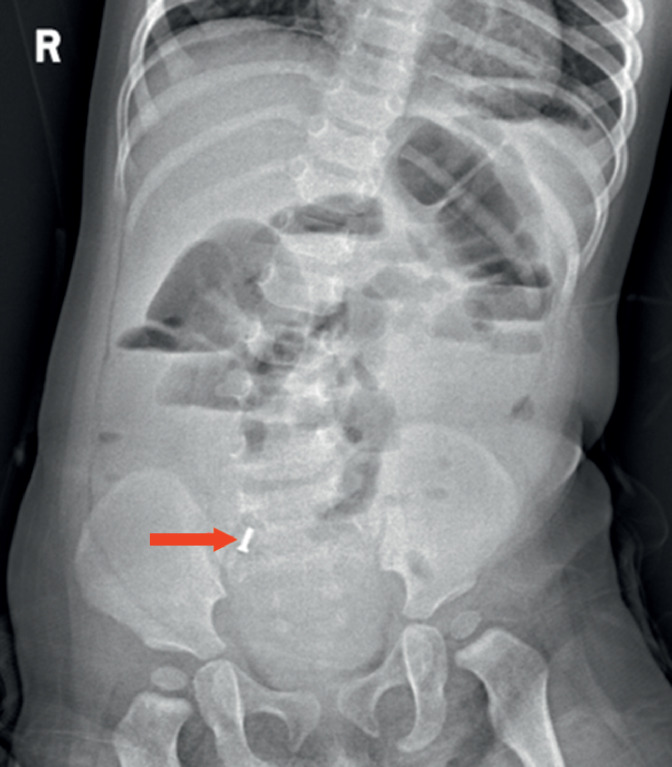
X-ray showed a screw (arrow) in the right lower quadrant in a 13-month-old girl.

**Fig. 2:**
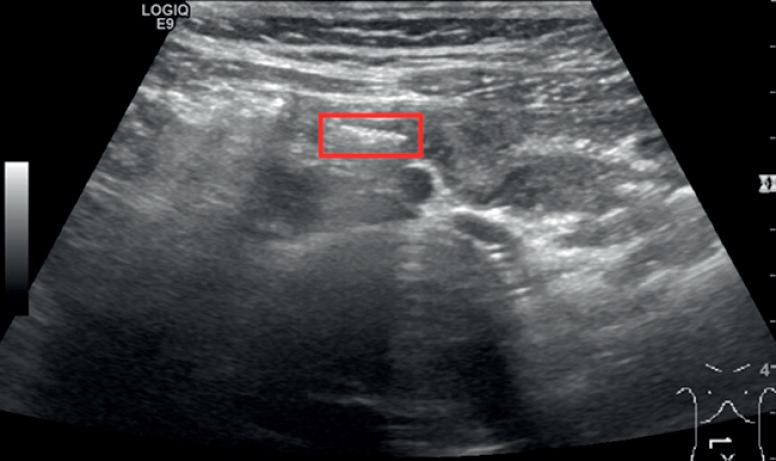
Ultrasound imaging of the appendix demonstrated the intraluminal position of the screw (red rectangle).

Endoscopic treatment was performed under anesthesia with endotracheal intubation. We used a colonoscope with a clear cap (H290I; Olympus, Tokyo, Japan) to intubate the cecum and observe the appendiceal orifice, which we found to be smooth. Subsequently, we inserted a disposable biliopancreatic duct imaging catheter (DPIC, D-000021494; Micro-Tech, Nanjing, China) into the appendiceal orifice and, after irrigation, found the screw deep in the appendix. Finally, we used a spiral stone extraction basket (CEB00000; Nanjing Weike Technology, China) to remove the screw ( Video 1 , Fig. 3 , Fig. 4 ). The patient had no postoperative complications and was discharged 2 days later.

**Fig. 3:**
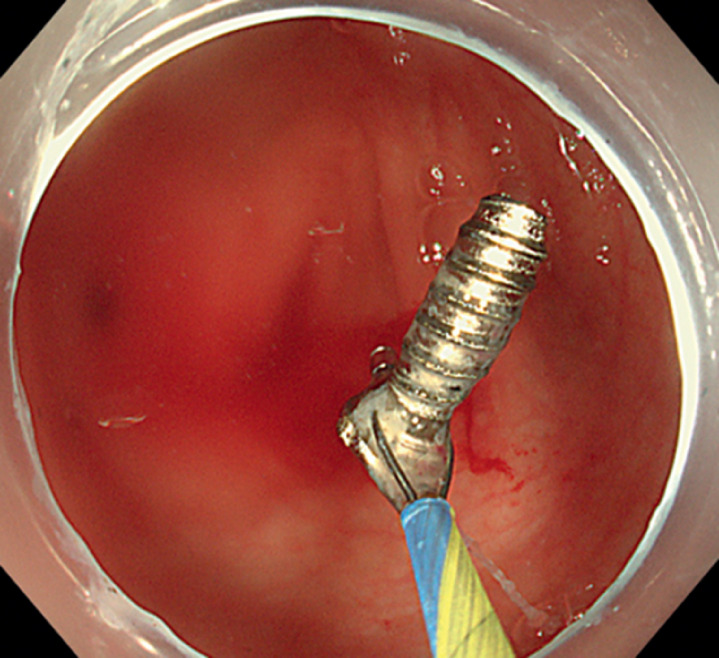
The screw was removed using a spiral stone-extractor basket.

**Fig. 4:**
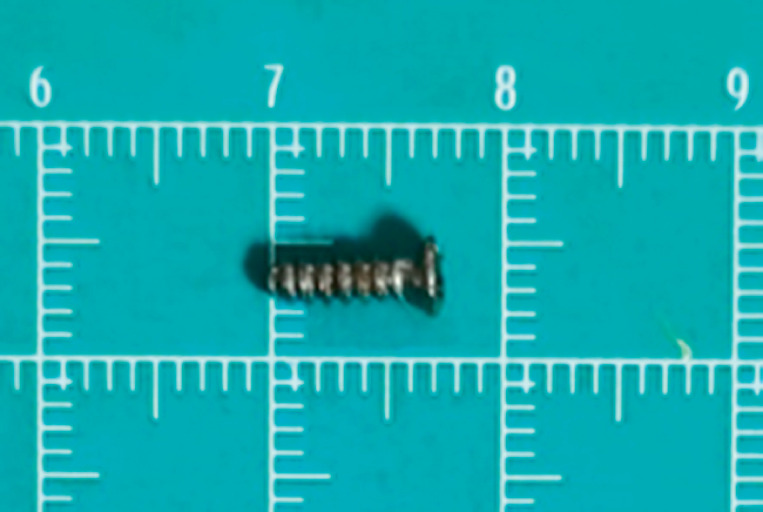
The removed screw.

Endoscopic removal of a foreign body (a screw) in the appendix of a 13-month-old girl.Video 1

Appendiceal foreign bodies in children are very rare. The largest series, reported by Fuller et al., showed that hair and plant material were the most common foreign objects found in the appendix 1 . Appendiceal foreign bodies in children are sometimes asymptomatic; however, they may lead to perforation, secondary appendiceal abscess and abdominal infection 2 3 . The appendiceal cavity in children is very narrow; it is difficult for foreign bodies to be expelled spontaneously once they enter into the appendix, except in a few cases 4 . To the best of our knowledge, our patient is the youngest and lightest patient in whom a biliopancreatic duct imaging system was used to enable endoscopic retrograde treatment for an appendiceal foreign body.

Endoscopy_UCTN_Code_TTT_1AQ_2AH

## Notes

Endoscopy E-Videos https://eref.thieme.de/e-videos
*E-Videos* is an open access online section of the journal *Endoscopy* , reporting on interesting cases and new techniques in gastroenterological endoscopy. All papers include a high-quality video and are published with a Creative Commons CC-BY license. Endoscopy E-Videos qualify for HINARI discounts and waivers and eligibility is automatically checked during the submission process. We grant 100% waivers to articles whose corresponding authors are based in Group A countries and 50% waivers to those who are based in Group B countries as classified by Research4Life (see: https://www.research4life.org/access/eligibility/ ). This section has its own submission website at https://mc.manuscriptcentral.com/e-videos .
